# Using Machine Learning–Based Approaches for the Detection and Classification of Human Papillomavirus Vaccine Misinformation: Infodemiology Study of Reddit Discussions

**DOI:** 10.2196/26478

**Published:** 2021-08-05

**Authors:** Jingcheng Du, Sharice Preston, Hanxiao Sun, Ross Shegog, Rachel Cunningham, Julie Boom, Lara Savas, Muhammad Amith, Cui Tao

**Affiliations:** 1 School of Biomedical Informatics The University of Texas Health Science Center at Houston Houston, TX United States; 2 School of Public Health The University of Texas Health Science Center at Houston Houston, TX United States; 3 Texas Children’s Hospital Houston, TX United States; 4 Baylor College of Medicine Houston, TX United States

**Keywords:** HPV vaccine, social media, misinformation, infodemiology, infoveillance, deep learning, Reddit, machine learning

## Abstract

**Background:**

The rapid growth of social media as an information channel has made it possible to quickly spread inaccurate or false vaccine information, thus creating obstacles for vaccine promotion.

**Objective:**

The aim of this study is to develop and evaluate an intelligent automated protocol for identifying and classifying human papillomavirus (HPV) vaccine misinformation on social media using machine learning (ML)–based methods.

**Methods:**

Reddit posts (from 2007 to 2017, N=28,121) that contained keywords related to HPV vaccination were compiled. A random subset (2200/28,121, 7.82%) was manually labeled for misinformation and served as the gold standard corpus for evaluation. A total of 5 ML-based algorithms, including a support vector machine, logistic regression, extremely randomized trees, a convolutional neural network, and a recurrent neural network designed to identify vaccine misinformation, were evaluated for identification performance. Topic modeling was applied to identify the major categories associated with HPV vaccine misinformation.

**Results:**

A convolutional neural network model achieved the highest area under the receiver operating characteristic curve of 0.7943. Of the 28,121 Reddit posts, 7207 (25.63%) were classified as vaccine misinformation, with discussions about general safety issues identified as the leading type of misinformed posts (2666/7207, 36.99%).

**Conclusions:**

ML-based approaches are effective in the identification and classification of HPV vaccine misinformation on Reddit and may be generalizable to other social media platforms. ML-based methods may provide the capacity and utility to meet the challenge involved in intelligent automated monitoring and classification of public health misinformation on social media platforms. The timely identification of vaccine misinformation on the internet is the first step in misinformation correction and vaccine promotion.

## Introduction

### Background

Human papillomavirus (HPV) infection is a highly prevalent sexually transmitted infection. HPV infections cause approximately 33,700 cases of cancer every year in the United States, including cervical, vaginal, penile, anal, and head and neck cancers [1,2]. Since 2006, a vaccine against the most common HPV subtypes has been available to prevent associated cancers and genital warts [3]. Despite undeniable evidence of its effectiveness, the HPV vaccine has been controversial among parents, which has contributed to vaccine hesitancy and even refusal [4] and to relatively low national rates of HPV vaccine initiation and series completion [5]. Resistance to the HPV vaccine has been a result of parents’ concerns about the vaccine’s effect on sexual behavior, because HPV is a sexually transmitted infection, and the safety of the vaccine, as well as inconsistent vaccine recommendations from health care providers [6].

A burgeoning antivaccine movement has affected overall vaccine coverage in the United States and contributed to a resurgence of vaccine-preventable diseases such as measles [7]. Vaccine hesitancy has been found to be driven mainly by concerns about vaccine safety and is propelled by misinformation circulated through social media [8]. The rapid growth of social media as an information channel has made it possible to quickly spread inaccurate or false information and create a platform for antivaccine campaigns to promulgate vaccine-related misinformation [9]. Participants in the antivaccine movement circulate antivaccine sentiments and misinformation through various internet channels and create demonstrable impact on individual and community health [10]. Experts in media communications have suggested that web-based misinformation is becoming unmanageable, even as concern increases about the damage it causes to consumer well-being [11]. Efforts to curtail the phenomenon, such as story-flagging and fact-checking tools, are not enough to suppress the advocates of misinformation [12,13] because the efficiency and scalability of these tools are limited, and misinformation is disseminated much faster and broader than true information.

Mitigation of medical and public health misinformation on social media is important; however, the sheer amount of information makes it challenging to identify these posts efficiently and accurately. Although social media is a convenient way for users to generate, share, receive, and comment on social content [14], there is a need for broad-scale, innovative methods to track and understand the spread of health misinformation on social media outlets [15].

Identifying vaccine-related misinformation presented on social media is an important first step in the timely curbing of the ongoing spread of vaccine misinformation. Given the large volume of social media posts and unique features of social media language (ie, incomplete sentences and misspellings), the use of automated methods for the identification of misinformation is challenging. However, machine learning (ML)–based approaches have been previously applied to identify misinformation on Twitter regarding controversial topic domains [16] and rumors regarding a range of topics [17]. ML involves the use of algorithms and statistical modeling that provide the ability to automatically conduct tasks and learn without using explicit programming [18]. Despite the utility of these ML approaches, there is a dearth of application to medical or health topics. To date, an ML-based system has tracked misinformation about the Zika virus on social media [19] and classified misinformation within specific health forums (eg, MedHelp) [20]. Although there have been efforts to develop ML for sentiment analysis on vaccine topics [21,22], to our knowledge, there is no prior work on *automated identification* of vaccine-related misinformation on social media. Deep learning (DL) is a subset of ML algorithms based on deep neural networks. Although DL has advanced ML algorithms in multiple tasks [23], the utility of DL regarding vaccine misinformation identification is still unclear.

### Objective

We report the utility of various conventional ML and DL algorithms to automatically identify and categorize misinformation on the HPV vaccine using posts on Reddit, a popular social media platform with more than 330 million monthly active users [24]. Reddit users are primarily anonymous young users below the age of 35 years, and more than half (54%) live in the United States [25]). Studies have revealed that young adults in the United States have low perceived susceptibility of contracting HPV [26], low health literacy pertaining to HPV and the HPV vaccine [27], and are more likely to seek health information on social media than other age demographics [28]. Table S1 in Multimedia Appendix 1 lists ML-related terms in the manuscript and their definitions.

## Methods

### Overview

**Figure 1:**
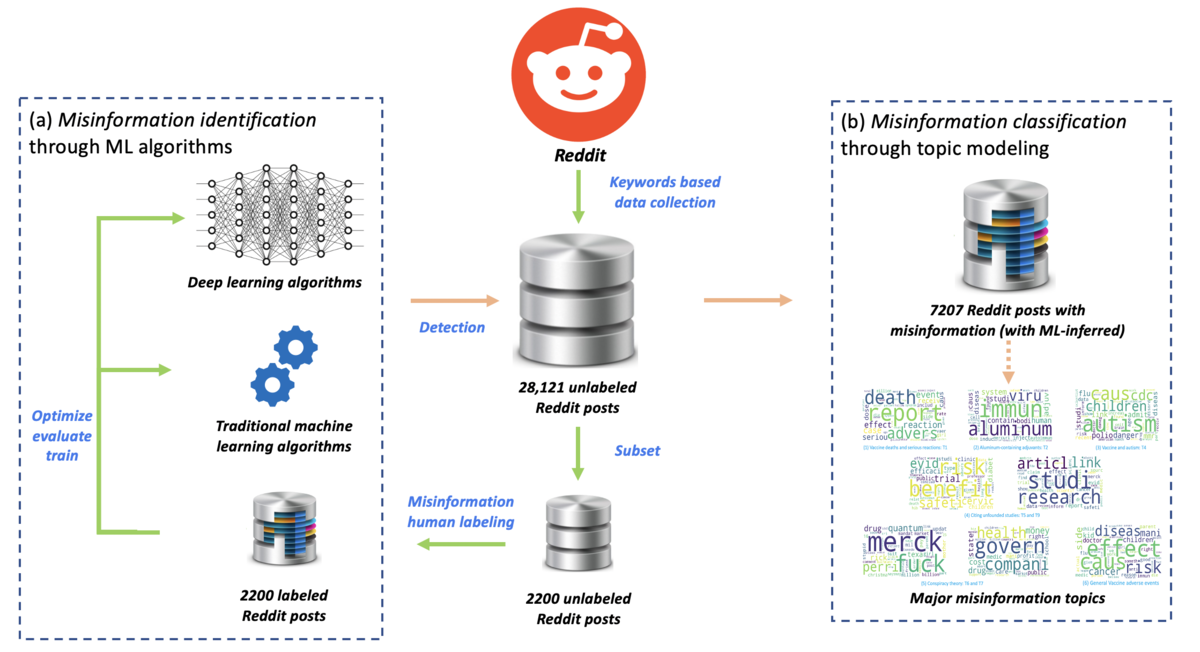
The overview of human papillomavirus misinformation identification and classification on Reddit. (a) Evaluation of machine learning–based misinformation identification and (b) topic modeling. ML: machine learning.

We used a hybrid approach for the identification and classification of HPV vaccine misinformation on Reddit (Figure 1). Our approach can be divided into two steps: (1) evaluation of ML algorithms for vaccine misinformation identification and (2) topic modeling on Reddit posts that contain vaccine misinformation (ML-inferred).

Reddit posts that contain HPV vaccine keywords were first collected, after which random subsets of the posts were labeled as containing *misinformation* or *nonmisinformation*. The labeled Reddit posts served as the gold standard corpus for the training and evaluation of various traditional ML and DL algorithms. The best algorithm, one that achieved the highest area under the receiver operating characteristic curve (AUC) [29], was then selected to infer the misinformation label for the remaining unlabeled Reddit posts. Finally, we applied topic modeling to the Reddit posts that were classified as misinformation to explore the major discussion topics and their prevalence.

### Data Set Collection and Labeling

We collected Reddit discussions related to HPV vaccination from 2007 to 2017 (N=28,121) using Pushshift [30]. Submissions (topic starters) and comments (responses to the topic) that contained the case-insensitive expressions of *cervarix* or *gardasil* or the combination of *hpv* or *papillomavirus* with *shot* or *vaccine* were collected.

We selected a random sample (simple random sampling) of the total collected Reddit posts (2200/28,121, 7.82%) and labeled the posts as misinformation or nonmisinformation. The purpose of this step was to build a gold standard corpus (ie, Reddit posts with their expert-assigned labels) that was used for the training and evaluation of the automated ML algorithms. The definition of vaccine misinformation was largely informed by the *Antivaccination Information* class of the Vaccine Misinformation Ontology (VAXMO) [31], a formal ontology to describe vaccine misinformation. Within the VAXMO, the *Antivaccination Information* class includes several subclasses, such as *Vaccine inefficacy*, *Alternative medicine*, *Civil liberties*, *Conspiracy theories*, *Falsehoods*, and *Ideological*. The random sample of the Reddit posts was used to develop a guideline through discussion among the annotators. A priori consensus was reached among 3 of the study annotators to combine the subclasses *Civil Liberties* and *Ideological* and to add two categories: *Vaccine recommendations* and *Other.* The resultant decision rules were that if a Reddit post contained one or more types of vaccine misinformation, it was considered an instance of misinformation (Textbox 1).

**Descriptions of types of vaccine misinformation.**

- Vaccine inefficacy: vaccine misinformation related to concerns about the lack of effectiveness of vaccines.
- Vaccine safety: vaccine misinformation related to concerns regarding safety issues and supposed harmful ingredients.
- Conspiracy theories: vaccine misinformation related to accusations of a cover-up, where regulatory bodies purportedly have information about vaccines that they are hiding from the public.
- Vaccine recommendations: vaccine misinformation related to vaccine recommendation or schedule.
- Civil liberties and ideologies: the encroachment on personal and parental legal rights or personal principles influencing individual opinions about antivaccine sentiment based on religion, morality, or other ideological reasons.
- Other: other types of vaccine misinformation or a mixed type of misinformation.

Furthermore, 3 study team members in the fields of biomedical informatics and public health (JD, SP, and HS) were involved in the annotation. The first 100 Reddit posts served as training, with the annotators independently annotating each post and then discussing each post and its annotation as a group. The training ended when the annotators achieved consensus (or took a decision based on a majority vote) on all the posts. After the annotation training, the remaining sampled Reddit posts were split among the 3 annotators for independent labeling. To examine the quality of the annotation, we selected 200 additional posts from the unlabeled Reddit posts, and JD, SP, and HS worked on these posts independently. We calculated the Cohen κ among the 3 annotators [32]. The total labeled Reddit samples were used as the basis of a gold standard corpus that was subsequently used for the training and evaluation of the automated ML algorithms.

### Misinformation Identification

#### Text Classification

Text classification is a fundamental task of natural language processing (NLP) which aims to classify the textual posts into predefined classes [33]. NLP is a subfield of artificial intelligence that allows computers to process and analyze natural language (ie, free text) data. We framed the identification of misinformation from Reddit posts as a binary text classification task. Each Reddit post was assigned one of two exclusive labels (ie, misinformation or nonmisinformation) within the automated ML-based algorithms (described below).

#### ML Algorithms

We evaluated 5 ML-based algorithms: 3 conventional and 2 DL algorithms. Traditional ML algorithms (ie, nondeep neural network–based algorithms) with feature engineering are widely used for text classification tasks. Altogether, 3 conventional ML algorithms were evaluated in this study: a support vector machine, logistic regression (LR), and extremely randomized trees. Support vector machines have been widely used in text classification tasks [34-36]. LR has achieved favorable performance on many task classification tasks as well but requires substantially less running time [37,38]. Extremely randomized trees is a tree-based ensemble method that has achieved favorable performances in our previous studies on social media text classification tasks [39,40]. Term frequency-inverse document frequency (TF-IDF) was adopted as the feature for these traditional ML algorithms. TF-IDF is a numerical statistic that assesses the relative importance of a word to a document in a corpus [41].

DL is a subset of ML algorithms. We evaluated 2 commonly used DL-based frameworks in this study: convolutional neural network (CNN) [42] and recurrent neural network (RNN) [43]. The effectiveness of traditional ML algorithms depends on task-specific feature engineering [44]. Deep neural networks can take advantage of pretrained word embedding to capture the semantics of words, which saves significant effort in feature engineering by domain experts [45]. DL algorithms have achieved state-of-the-art performance on many text classification tasks [46-49].

As there are frequent occurrences of incorrect spelling in social media posts, both the evaluated DL algorithms contained a character layer and a word-embedding layer to map both in-vocabulary (ie, correctly spelled) and out-of-vocabulary (ie, incorrectly spelled) words to high-dimensional vectors to represent their semantics. GloVe (Global Vectors for Word Representation) embedding (ie, glove.840B.300d) [50] was used to initialize the weights in the word-embedding layer. The CNN model takes word-level embedding as input and feeds it to convolution and max-pooling layers, a fully connected layer and a softmax layer, respectively, for classification [42]. The RNN model follows an architecture that is similar to that of the CNN model by replacing convolution and max-pooling layers with bidirectional long short-term memory layers and attention layers.

More specifically, for both the CNN and RNN models, the learning rate was set at 0.01, the batch size was 64, and the number of epochs was set at 100. The length of the character embedding was set at 50 for both models. For the CNN model, the filter sizes were 1, 2, and 3, and the number of filters was 2048; for the RNN model, the hidden dimension of the long short-term memory unit and attention layer was set to 128. The dropout probability was 0.2 for both models. The model that achieved the best AUC value on the validation set was selected for testing and prediction.

#### Experiment Settings and Evaluation

The gold standard posts (ie, Reddit posts with expert-assigned labels) were randomly split into train, validation, and test sets in a ratio of 7:1:2. We adopted spaCy *tokenization* [51] to split the post text into separate words, remove punctuations, and convert words and letters in uppercase to lowercase. Sequentially, the train set was used to *train* the algorithms, the validation set was used for hyperparameter selection, and the test set was used to evaluate the performance of the models. To account for imbalance in label distribution, the criterion of reference was the degree of specificity as measured by the optimal AUC. The algorithm with the highest AUC value was selected for the inference of vaccine misinformation from unlabeled Reddit posts.

We further plotted the precision and recall curves of the best-performing model (ie, the CNN model) and selected the optimal cutoff (based on the highest *F*_1_ score) of the algorithm to identify vaccine misinformation in Reddit posts. Precision was defined as the fraction of misinformation posts identified by the labelers among the fraction of misinformation posts identified by the classifier. Recall was defined as the fraction of misinformation posts identified by the labelers that were retrieved by the classifier. The *F*_1_ score is a harmonic mean of precision and recall. The cutoff that led to the best *F*_1_ score for the CNN model was selected. The model was applied to identify vaccine misinformation–related Reddit posts in the remaining unlabeled Reddit corpus.

### Misinformation Topic Model

The ML and DL algorithms described above can potentially be used to automatically identify Reddit posts with misinformation, but they do not categorize the types of misinformation. We adopted a topic model algorithm (ie, Biterm Topic model [BTM]) [52], and we implemented the code from a GitHub repository [53]) to identify and visualize major topics from the misinformation in Reddit posts. Topic models are a type of statistical model designed to cluster the abstract *topics* that occur in a collection of documents. After using the best-performing ML algorithm to identify Reddit posts that contain misinformation, we then applied the BTM to these posts. We performed stemming for each word to remove morphological affixes (eg, *dies* to *die* and *denied* to *deni*). The number of topics is a hyperparameter for the BTM, which determines the number of topics that will be generated. We evaluated 5, 10, and 20 as the number of topics and selected 10 through a manual review of the topics and associated posts. We then manually reviewed these topics, associated words, and posts to further identify relevant topics associated with vaccine misinformation. The BTM also outputs the prevalence of each identified topic. Word clouds were then adopted to provide a graphic representation of these topics, where the size of each word is proportional to its probability of appearing in posts about that topic [54]. To examine the association of the identified topics, we further performed network analysis among these topics.

### Ethics Approval and Consent to Participate

This study received institutional review board exemption from the committee for the protection of human subjects at the University of Texas Health Science Center at Houston. The reference number is HSC-SBMI-20-0151.

## Results

### Misinformation Annotation

In total, 28,121 Reddit posts were collected from 2007 to 2017 from more than 16,633 unique users. The statistics of these posts as well as their distributions in subreddits (ie, user-created discussion boards where posts are organized by a subject) are shown in Table 1. There was an increasing trend of HPV vaccine–related discussions (in terms of both the number of posts and number of unique users) during the study period. There were 207,651 upvotes (a user likes the post) and 10,700 downvotes (a user does not like the post) for these posts. Of the 28,121 posts, we manually labeled 2200 (7.82%) randomly selected posts. We measured the annotation agreement by calculating the Cohen κ among the 3 annotators: 0.5578 for JD and HS, 0.5216 for JD and SP, and 0.4685 for HS and SP. The agreement scores are considered moderate according to El Eman [32], which indicates a good quality of our gold standard. Among these 2200 posts, 396 (18%) were annotated as vaccine misinformation, whereas 1804 (82%) were annotated as nonmisinformation. The highly imbalanced label distribution created barriers to achieving high performance for the classification algorithms.

**Table 1:** The statistics of the human papillomavirus Reddit posts corpus. For statistics regarding Reddit users, we removed the posts if the accounts were unavailable.

Year	Total posts	Total upvotes	Total downvotes	Total unique users	User posts distribution, mean (SD)	Most frequent subreddits^a^ (top 3)	Subreddit post distribution, mean (SD)
2007	15	51	1	10	1.00 (0.00)	Reddit, 11; politics, 3; science, 1	5.00 (5.29)
2008	172	335	35	100	1.34 (1.32)	Reddit, 57; science, 54; health, 23	11.47 (18.88)
2009	414	1563	206	249	1.39 (0.93)	Science, 81; AskReddit, 51; Reddit, 47	14.28 (18.96)
2010	546	1655	155	346	1.33 (0.79)	AskReddit, 95; sex, 83; TwoXChromosomes, 72	12.13 (22.37)
2011	2156	12,711	927	1382	1.37 (1.47)	Politics, 298; TwoXChromosomes, 207; AskReddit, 203	19.42 (48.48)
2012	2457	12,812	739	1641	1.32 (1.34)	AskReddit, 457; TwoXChromosomes, 308; sex, 221	13.96 (48.60)
2013	3864	26,623	1416	2540	1.39 (2.44)	Science, 490; AskReddit, 375; sex, 297	15.97 (51.53)
2014	3488	21,562	1581	2348	1.39 (2.62)	Sex, 325; AskReddit, 292; science, 291	10.67 (34.81)
2015	4714	35,801	1761	3383	1.38 (1.61)	News, 378; science, 357; AskReddit, 347	11.67 (41.28)
2016	4417	38,123	1436	3137	1.39 (1.43)	AskReddit, 378; TwoXChromosomes, 262; sex, 255	10.44 (34.25)
2017	5878	56,415	2443	3752	1.56 (9.09)	AskReddit, 446; sex, 402; news, 387	11.28 (40.95)

^a^Numbers included indicate counts.

### Misinformation Detection and Classification

**Figure 2:**
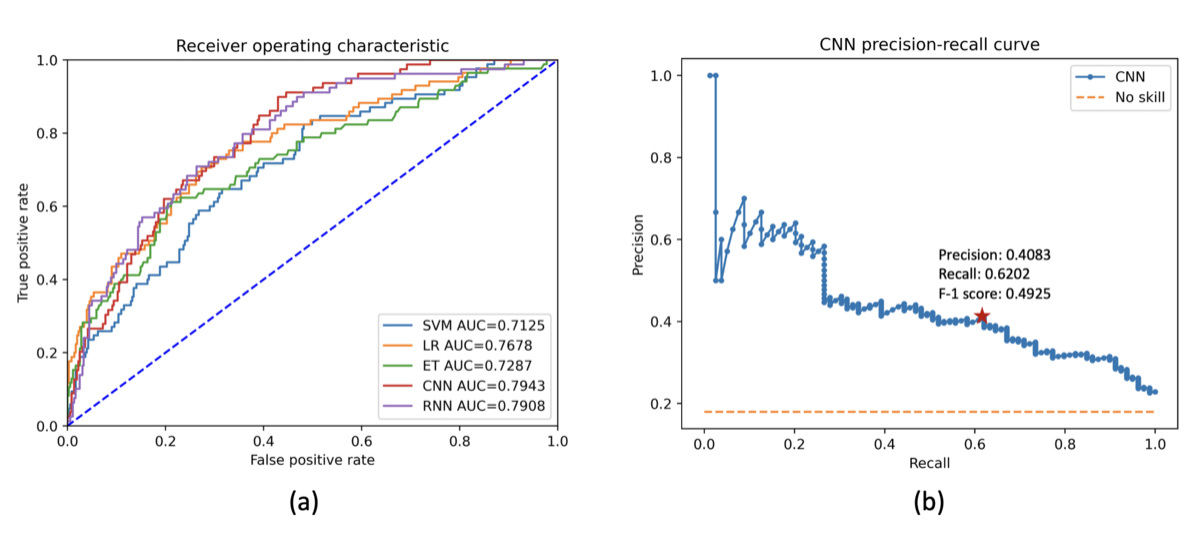
The performance of machine learning algorithms on human papillomavirus misinformation identification. (a) Receiver operating characteristic and (b) convolutional neural networks precision-recall curves. AUC: area under the curve; ET: extremely randomized trees; CNN: convolutional neural networks; LR: logistic regression; RNN: recurrent neural network; SVM: support vector machines.

The LR algorithm demonstrated the highest AUC value (0.7678) among the 3 traditional ML algorithms used to identify vaccine misinformation in the Reddit posts (Figure 2). Both DL algorithms (CNN and RNN) achieved higher AUC values than the traditional ML algorithms. The CNN model slightly outperformed the RNN model (0.7943 vs 0.7908) in the identification of misinformation. The CNN model with the optimal cutoff was applied to classify the Reddit posts that contained vaccine misinformation. The precision and recall curves of the CNN model are shown in Figure 2. The optimal cutoff led to a precision of 0.4083, a recall of 0.6202, and an *F*_1_ score of 0.4925. Together with 1.41% (396/28,121) of the Reddit posts that were manually annotated as misinformation, 25.63% (7207/28,121) of the random subset of posts were classified as vaccine misinformation.

Topic modeling generated 10 topics from 7207 Reddit posts that were classified as vaccine misinformation. Through qualitative analysis of these 10 algorithm-identified topics and a review of their relevant Reddit posts, we condensed them into 7 (6 major topics + *Other*) topics. The word clouds for the 6 identified misinformation topics are shown in Figure S1 of Multimedia Appendix 1. The 6 major topics, the percentage of posts assigned to the topic, and post examples are listed in Table 2.

**Table 2:** The major topics of misinformation identified by topic modeling (n=7207).

Misinformation topic	Prevalence, n (%)	Explanation	Examples (excerpts)
General vaccine adverse events	2672 (37.07)	Promotion of general misinformation regarding the safety of HPV^a^ vaccine	“The HPV vaccine is unnecessary and unsafe.” “The HPV vaccination causes retardation.”
Conspiracy theory	1072 (14.87)	Propagation of conspiracy theories about HPV vaccine and fraud by the government and large pharmaceutical companies (eg, Merck)	“Rick Perry signed an executive order trying to mandate Gardasil vaccines for young girls. He did this to make money for his buddies at Merck.” “The HPV vaccine is a joke pressed on schools and administrations by greedy pharmaceutical corporations.”
Citing unfounded studies	989 (13.72)	This type of misinformation can be very misleading because it tends to cite and interpret *scientific* studies from sources that are not scientifically peer reviewed	“Flawed safety study on HPV vaccine triggers butterfly effect - leaked e-mails reveal who suppressed info on dangerous particles in vaccine” “We find that HPV vaccine clinical trials design, and data interpretation of both efficacy and safety outcomes, were largely inadequate”
Vaccine deaths and serious reactions	520 (7.21)	Propagation of HPV vaccine–induced death and serious adverse reactions	“For instance, the HPV vaccine has caused children to die.” “The shots have killed women. The adverse event reports from the FDA^b^ about HPV vaccination read like a litany of horrors.”
Aluminum-containing adjuvants	456 (6.33)	Promoting misinformation on safety issues of aluminum-containing adjuvants in vaccines	“Another good keyword is for Gardasil adverse drug reactions. That’s how I found this: ‘each 0.5-ml dose of the vaccine contains approximately 225 mcg of aluminum (as amorphous aluminum hydroxyphosphate sulfate adjuvant)’ This study clearly shows that aluminum found in vaccines can cause neurologic damage.” “Brain damage and autoimmune diseases can be caused by aluminum adjuvants. Aluminum adjuvant is in the HPV vaccine.”
Vaccine and autism	198 (2.75)	Promoting misinformation on the discredited links between vaccine and autism	“The HPV vaccine causes autism” “Vaccine court awarded millions to two children with autism. CDC^c^ report creates controversy for Merck’s Gardasil vaccine”

^a^HPV: human papillomavirus.

^b^FDA: Food and Drug Administration.

^c^CDC: US Centers for Disease Control and Prevention.

### Misinformation Network Analysis

We further analyzed the network among the identified topics. For each Reddit post, we identified the 2 most associated topics (ranked by probability generated by the BTM). We assume that these top 2 topics were linked for that post, which was considered an undirected edge in the network. Figure S2 in Multimedia Appendix 1 shows the misinformation topic network among these 7 topics. The size of the circle for each topic is proportional to the degree of associations of the topic (ie, the number of connections with other topics). The width of the edge is proportional to the number of connections between the 2 topics.

## Discussion

### Principal Findings

In this study, we evaluated the use of different ML-based approaches to analyze Reddit discussions related to HPV vaccine misinformation. The CNN and RNN algorithms improved the AUC value compared with the traditional ML algorithms. A BTM was adopted to further explore the major topics related to vaccine misinformation discussions. Overall, 6 major topics related to HPV vaccine misinformation, including *Vaccine death and serious reactions* and *Aluminum-containing adjuvants* were identified. The *Vaccine adverse effect*, which refers to general misinformation regarding safety issues, is the most prevalent topic within HPV vaccine misinformation.

The highest proportion of vaccine misinformation content on Reddit identified with our approach concerned general vaccine adverse effects (2672/7207, 37.07%), followed by content about vaccine conspiracy theories (1072/7207, 14.87%). These results are consistent with previous analyses of social media–based vaccine misinformation, which found that inaccuracies about vaccine knowledge and risk (37.9%) made up most of the social media posts with negative vaccine sentiments [55]. The same small study also found that 13.8% of the negative posts about vaccines included distrust of government and pharmaceutical companies, which closely mirrors our findings in a larger sample from Reddit.

We further analyzed the top subreddits among the posts that ML inferred as containing misinformation and nonmisinformation. The subreddits that contain the most misinformation-related posts include *science* (n=653), *AskReddit* (n=604), *conspiracy* (n=593), and *politics* (n=397). On the contrary, the subreddits that contain the most nonmisinformation-related posts include *AskReddit* (n=2040), *sex* (n=1994), *TwoXChromosomes* (n=1652), and *science* (n=1385). Besides general popular subreddits such as *science* and *AskReddit*, misinformation tends to cluster in subreddits such as *conspiracy* and *politics*. There is an increasing trend of discussing HPV vaccine–related topics on Reddit from 15 posts in 2007 to 5878 posts in 2017. There is a decreasing trend in the proportion of misinformation over time on Reddit. The proportion of misinformation ranged from 41.8% (72/172; in year 2008) to 53% (8/15; in year 2007) during the period 2007 to 2009, whereas it ranged from 22.84% (1009/4417; in year 2016) to 33.85% (730/2156; in year 2011) during the period 2010 to 2017. The decrease could be a result of the continuous promotion efforts made by public health professionals, as well as an increase in internet verification skills among users.

The results of our network analysis of the 6 identified vaccine misinformation topics (in addition to *Other*) further reinforce our findings and demonstrate the strength of the connectedness of each topic. Although general concerns about the safety of the vaccine emerged as the main source of hesitancy regarding HPV vaccination, the network analysis indicates that the other prominent topics identified, such as the presence of conspiracy theories, may also be rooted in fears about the side effects of the vaccine. Mere exposure to beliefs that the government and pharmaceutical companies gain or profit from mass vaccination through deception or at the consumers’ expense, has strong negative effects on attitudes about the safety and effectiveness of vaccines, consequently affecting choices about whether to vaccinate [56].

Of note, the annotators anecdotally observed that the Reddit posts identified in this study did not seem to be connected to any organized movements; rather, they were by single users advocating their personal views. A potential method to combat these misinformed messages once identified is to counter them with an organized campaign, composed of factual, evidence-based messages, that does not acknowledge disinformation. As other studies have noted, acknowledging and deferring to web-based disputes related to vaccines may cause health information seekers to doubt established evidence regarding vaccine efficacy and safety [57]. In addition, it has been found that attempting to correct misinformation directly often reinforces the sentiments of those holding strong antivaccine views [58].

To the best of our knowledge, this is one of the early efforts to explore the use of *automated* ML algorithms (eg, ML and NLP) to identify and classify HPV vaccine misinformation in social media discussions. We chose the HPV vaccine as the use case for our analysis, but the proposed methodology framework can also be applied to other types of vaccines or other pertinent health-related topics. The ML-based framework is also scalable to big social media data. Our work could assist policy makers and the industry to accurately understand and address the spread of health misinformation on social media. The methodology framework developed in this study is generalizable to other social media platforms such as Twitter and can be used to identify misinformation in both retrospective and real-time social media feeds. The use of this methodology could be incorporated into social media platforms dedicated to curbing the spread of health-related misinformation on these sites, although the ethical ramifications of such restrictions should be taken into consideration.

### Limitations and Future Work

This study should be interpreted in light of limitations and future research needs. Given the unique features of social media language, the accurate identification of misinformation is a very challenging task. The best algorithm achieved an AUC value of 0.7943, and there is some room for improving this performance. Our current ML classifier has a higher recall than precision (0.6202 vs 0.4083). This means that the classifier tends to label both misinformation and nonmisinformation as misinformation. In a real-world scenario, the classifier may serve as a tool to prescreen misinformation, and more rigorous fact-checking methods (eg, human checking) would be needed to label true misinformation posts. The high imbalanced label distribution (ie, only 396/2200, 18%) of the posts were labeled as misinformation in the gold standard corpus) hurt the ML algorithm because most of the ML algorithms used for classification were designed based on the assumption of an equal number of examples for each class. Imbalanced label distribution results in models that have poor predictive performance, specifically for the minority class (eg, misinformation in our case) [59]. As we further refine and expand the gold standard corpus, which is critical for the evaluation and training of ML algorithms, we expect the performance to improve. In addition, we will explore the use of data augmentation techniques [60] and random oversampling methods [61,62] to alleviate the issues caused by imbalanced label distribution. Other emerging advanced DL algorithms such as Bidirectional Encoder Representations from Transformers (BERT) [63] hold promise for improved performance. In addition, we performed annotation at the level of Reddit posts, which may have sacrificed precision. A single Reddit post often contains multiple sentences, allowing a mix of misinformation and nonmisinformation to exist in a single post. Therefore, a Reddit post annotated as *misinformation* could also contain evidence-based facts. Future research can establish the effect of annotation and classification at the sentence level to improve the precision of misinformation identification. In addition, the identification of *abstract topics* from topic modeling is a semiautomated process combined with expert review. However, topic assignment and summarization may be subjective and suffer from biases as well. In future, we can explore the use of supervised algorithms for more precise topic discovery.

### Conclusions

Our ML-based approaches demonstrated efficacy in the automated identification and classification of HPV vaccine misinformation in discussions on the social media platform Reddit. The large quantity of web- and social media–based medical and public health information available may make it difficult for those with low health and web literacy to navigate and find authentic and evidence-based information. Although our ML algorithm does not solve the problem of health and vaccine misinformation single-handedly, we provide an innovative stepping stone that may bridge multiple approaches for combating this invasive and growing public health concern. The accurate and timely understanding of vaccine misinformation on social media can assist vaccine promotion campaigns to prevent such information from misleading the vulnerable public. Our methodology could also be applied to other social media platforms such as Twitter, although new labeled data would be necessary.

## Appendix

Multimedia Appendix 1 Supplemental tables and figures.

## Abbreviations

AUC: area under the receiver operating characteristic curve
BERT: Bidirectional Encoder Representations from Transformers
BTM: Biterm Topic model
CNN: convolutional neural network
DL: deep learning
GloVe: Global Vectors for Word Representation
HPV: human papillomavirus
LR: logistic regression
ML: machine learning
NLP: natural language processing
RNN: recurrent neural network
TF-IDF: term frequency-inverse document frequency
VAXMO: Vaccine Misinformation Ontology
