# Supplementary material for: Using Machine Learning–Based Approaches for the Detection and Classification of Human Papillomavirus Vaccine Misinformation: Infodemiology Study of Reddit Discussions
Source: J Med Internet Res. 2021 Aug 5;23(8):e26478. doi: 10.2196/26478 (PMC8380585; doi:10.2196/26478)
Supplement: Multimedia Appendix 1 [file jmir_v23i8e26478_app1.docx]

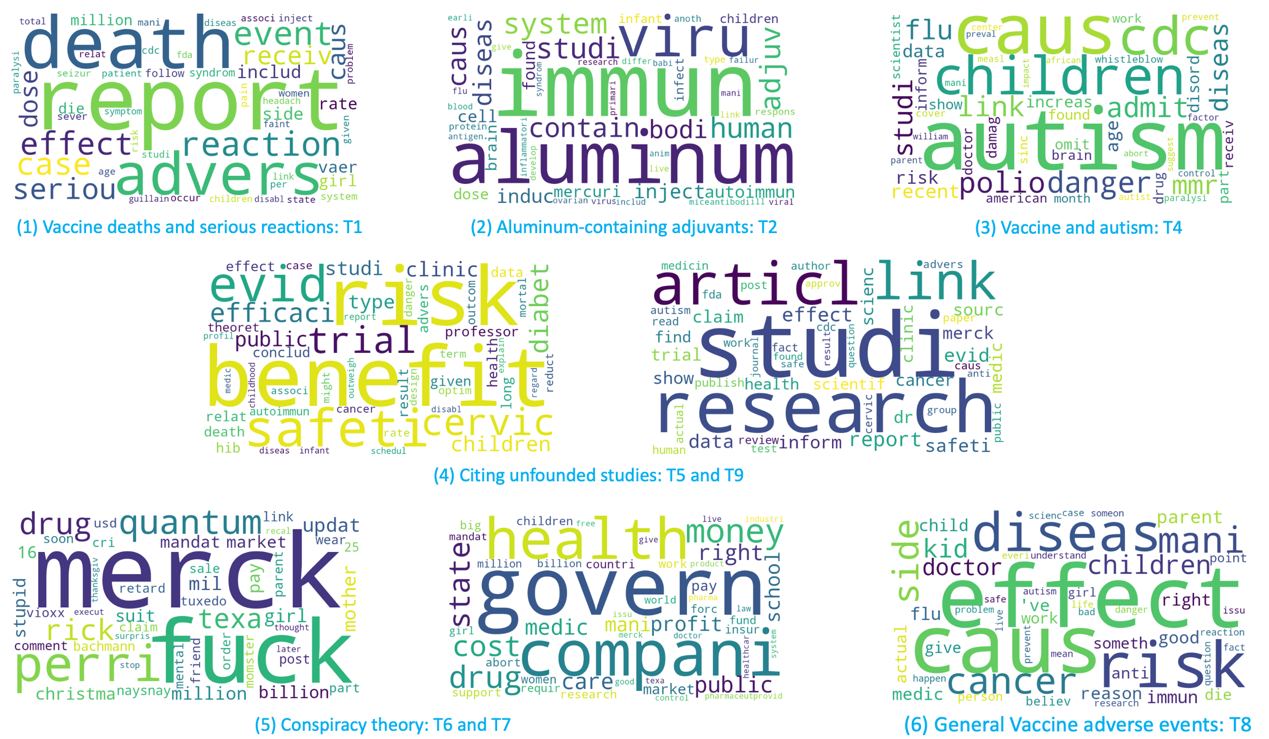


Figure S1. The word clouds of major topics of HPV vaccine misinformation


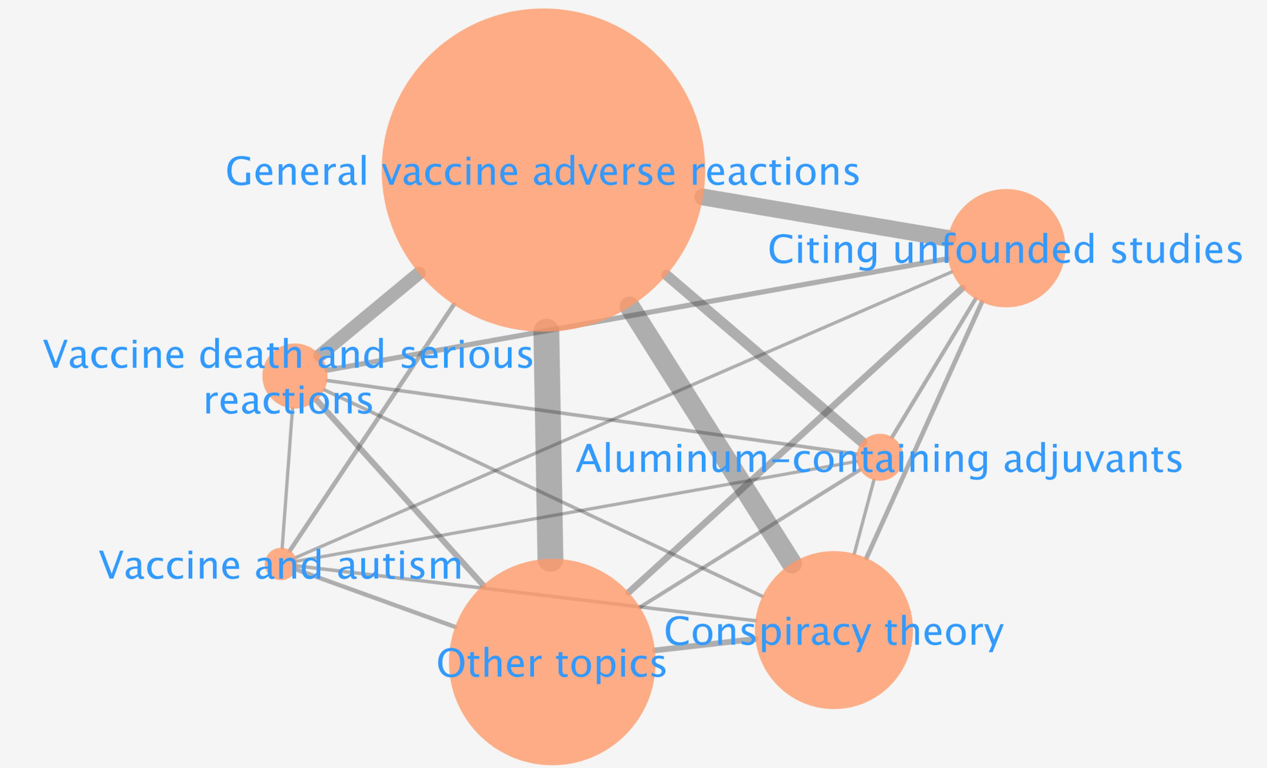


Figure S2. HPV vaccine misinformation topic network

Table S1 Definition of Terms.

| Term | Definition |
| --- | --- |
| Natural language processing (NLP) | NLP is a field of artificial intelligence (AI) that understands natural language through computers. |
| Gold standard corpus | Gold standard corpus is a human-annotated trustworthy dataset that is used for training and evaluation of computational algorithms (e.g., machine-learning algorithms). |
| Machine learning (ML) | ML is an application of AI that provides computers with the ability to learn and improve through experience without being explicitly programmed. |
| Supervised learning | Supervised learning is a type of ML that learns a function that maps an input to an output based on example input-output pairs in the labeled dataset. |
| Support vector machine (SVM) | SVM is a supervised ML model that finds a hyper-plane in an N-dimensional space (N is the number of features) that distinctly classifies the data points. |
| Logistic regression (LR) | LR is a supervised ML model that uses a logistic function to predict the probability of a target output. |
| Extremely randomized trees (ET) | ET is a supervised ML model that is based on a tree-based ensemble method, consisting of randomizing strongly both attribute and cut-point choice while splitting a tree node. |
| Term frequency-inverse document frequency (TF-IDF) | TF-IDF is a numerical statistic that is intended to reflect how important a word is to a document in a corpus. |
| Deep learning (DL) | DL is a subfield of ML that is based on artificial neural networks algorithms. |
| Convolutional neural network (CNN) | CNN is a DL model that is comprised of a number of convolutional and subsampling layers that are optionally followed by fully connected layers. |
| Recurrent neural network (RNN) | RNN is a DL model that contains loops within the network, which allows information to be stored. |
| Topic model | Topic model is a type of statistical model for discovering the abstract “topics” that occur in a collection of documents. Typical algorithms include latent Dirichlet allocation (LDA) and latent semantic analysis (LSA). |
| Biterm topic model (BTM) | BTM is a variant of topic model that learns the topics by directly modeling the generation of word co-occurrence patterns (i.e., biterms) in a collection of documents. |
| Area under the receiver operating characteristic curve (AUC) | A receiver operating characteristic (ROC) curve is a graph that shows the performance of a classification model at all classifying thresholds. Two parameters are plotted in the ROC curve, including the true positive rate and false positive rates. AUC measures the entire two-dimensional area underneath the entire ROC curve. AUC is preferred for binary classification when the label distribution in the test set is imbalanced. |
